# Criteria of radiological diagnosis for neonates with hypochondroplasia

**DOI:** 10.1186/1687-9856-2015-S1-O24

**Published:** 2015-04-28

**Authors:** Keisuke Nagasaki, Tomoko Saito, Masaki Takagi, Tomonobu Hasegawa, Gen Nishimura

**Affiliations:** 1 Niigata University Graduate School of Medicine and Dental Sciences, Niigata, Japan; 2 Tokyo Metropolitan Children’s Medical Center, Tokyo, Japan; 3 Keio University School of Medicine, Tokyo, Japan

## Introduction

The diagnosis of hypochondroplasia (HCH) is hampered by absence of radiological criteria relevant to the age-dependent manifestations, particularly those in the neonatal period. This work deals with the radiological features in hypochondroplastic neonates with a FGFR3 mutation, including quantitative measurement that facilitates the definitive diagnosis. We propose the radiological criteria for HCH in the neonatal period.

## Patients and methods

Subjects included six HCH neonates with FGFR3 mutations and 30 control subjects, in whom radiological examination was available as a neonate. The following findings were evaluated: 1) short ilia, 2) squared ilia, 3) short greater sciatic notch, 4) horizontal acetabula, 5) short femora, 6) stubby femora, 7) metaphyseal flaring, 8) lumboscaral interpediculate distance narrowing, and 9) oval radiolucency in the proximal femora.

## Results

All measurement parameters for short ilia, short greater sciatic notch, horizontal acetabula, short femora and stubby femora (parameters 1, 3, 4, 5, and 6) were statistically different between HCH and control, while the other parameters 2, 7, and 8 were not. Based on these results, we tentatively made the criteria and scoring system for the diagnosis of HCH. The major criteria that are given a score of 2 comprise parameters 1, 3, and 6, whose distribution was not overlapped between HCH and control. The minor criteria that are given a score of 1 point comprise parameters 4 and 5, and 9, because the parameter 4 was overlapped in distribution between HCH and control, the parameter 5 is a non-specific finding, and the parameter 9 is subjective in assessment. We presumed that a total score of 6 points or higher warrant a diagnosis of HCH.

## Conclusion

HCH was clearly distinguishable from normal infants assessing the skeletal findings of the ilia and proximal femora on neonatal roentgenograms. We compiled a set of diagnostic criteria for the early diagnosis of hypochondroplastic neonates.

